# Ochratoxin A in food commodities: A review of occurrence, toxicity, and management strategies

**DOI:** 10.1016/j.heliyon.2024.e39313

**Published:** 2024-10-12

**Authors:** Joel Cox Menka Banahene, Isaac Williams Ofosu, Bernard Tawiah Odai, Herman Erick Lutterodt, Paul Ayiku Agyemang, Williams Otoo Ellis

**Affiliations:** a Department of Food Science and Technology, Kwame Nkrumah University of Science and Technology, Kumasi, Ashanti, Ghana; b Research Department, Quality Control Company Limited–Ghana Cocoa Board, Tema, Greater Accra, Ghana; c Radiation Technology Centre–BNARI, Ghana Atomic Energy Commission, Kwabenya, Accra, Ghana

**Keywords:** Ochratoxin A, Food commodities, Public health, Economy, Prevention & control

## Abstract

Ochratoxin A (OTA) is a potent mycotoxin produced by species of Aspergillus and Penicillium that contaminate agricultural products and pose significant health risks to both humans and animals. This review examines the mechanisms of OTA toxicity, its occurrence in various food commodities, and the implications for public health and trade. Literature pertaining to OTA was sourced from Google Scholar, covering the period from 2004 to 2024. OTA exposure is linked to multiple adverse health effects, including teratogenicity, immunotoxicity, and hepatotoxicity, with a primary impact on kidney function, and it is classified as a possible human carcinogen (Group 2B). Its toxic effects are attributed to several mechanisms, including lipid peroxidation, inhibition of protein synthesis, DNA damage, oxidative stress, and mitochondrial dysfunction. Notable findings included the presence of OTA in 46.7 % of cocoa products in Turkey, 32 % of cocoa samples in Côte d’Ivoire exceeding the OTA threshold of 2 μg/kg, and 91.5 % of ready-to-sell cocoa beans in Nigeria testing positive for OTA. Coffee beans are particularly susceptible to OTA contamination, which underscores the need for vigilant monitoring. Additionally, OTA contamination impacts agricultural productivity and food safety, leading to significant economic consequences, particularly in regions reliant on exports, such as cocoa and coffee. Several countries regulate the OTA levels in food products to safeguard public health. However, these regulations can impede trade, particularly in countries with high levels of contamination. Balancing regulatory compliance with economic viability is crucial for affected nations. Current strategies for managing OTA include improved agronomic practices, such as the use of biocontrol agents for pest management, enhanced storage conditions to prevent mould growth, and the implementation of detoxification techniques to reduce OTA levels in food products. Despite these strategies, OTA remains a significant threat to public health and the agricultural economy worldwide. The complexity of contamination in food products requires robust prevention, control, and management strategies to mitigate its impact. Continuous research and regulatory initiatives are essential for safeguarding consumers and ensuring food safety.

## 1 Introduction

Annual crop yields demonstrate a downward trend, and according to estimates by the Food and Agriculture Organization (FAO), mycotoxin contamination affects approximately 25 % or more of food crops annually [1]. Mycotoxins produced by filamentous fungi have been extensively studied. However, it is important to recognize that only a specific subset of mycotoxins poses significant challenges to food safety [2,3]. Among these mycotoxins, those produced by *Aspergillus*, *Penicillium*, and *Fusarium* species warrant particular attention [4]. Notably, ochratoxin is a major mycotoxin produced by *Aspergillus* and *Penicillium* species [[5], [6], [7]]. The ochratoxin family encompasses more than 20 subtypes, including ochratoxin A (OTA), ochratoxin B (OTB), ochratoxin C (OTC), non-amide ochratoxin α (OTα) of OTA and OTC, non-amide ochratoxin β (OTβ) of OTB, and hydroxylated OTA and OTB (4R-OH OTA, 4S-OH OTA, 4-OH OTB, 10-OH OTA) [8]. Although these ochratoxin subtypes share a common structural similarity, their varying toxicities underscore the importance of distinguishing OTA as the most toxic member. OTA frequently contaminates a wide-range of food products, including cereals, cocoa, coffee, dried fruits, spices, wine and beer [[9], [10], [11], [12], [13], [14], [15]]. Numerous studies have established that OTA exhibits a range of toxic effects, including nephrotoxicity, hepatotoxicity, teratogenicity, neurotoxicity, carcinogenicity, genotoxicity, and immunotoxicity [[16], [17], [18], [19], [20], [21]].

Although complete elimination of OTA from the food chain is highly desirable, this remains a formidable challenge. However, there are effective practices within the realms of agriculture, manufacturing, and storage that can mitigate OTA contamination [22]. Inadequate toxicological and exposure assessment data hinder the comprehensive evaluation of human health risks associated with OTA consumption [23]. However, the International Agency for Research on Cancer (IARC) classifies OTA as Group 2B, indicating it is possibly carcinogenic to humans [24]. Additionally, the National Toxicology Program (NTP) identifies OTA as the most potent renal carcinogen in animal species [25].

Regulatory bodies, such as the Joint FAO/WHO Expert Committee on Food Additives (JECFA) and European Food Safety Authority (EFSA), have established provisional tolerable weekly intake (PTWI) levels for OTA: 120 ng/kg body weight and 100 ng/kg body weight, respectively, based on toxicological evaluations [26,27]. The European Union has set maximum limits for OTA in various food products: 5 μg/kg in cereals; 3 μg/kg in cereal-processed products; 5 μg/kg in roasted, ground, and instant coffee; 3 μg/kg in cocoa powder; and 2 μg/kg in wine.

OTA-contaminated foods pose serious health risks to consumers [28]. Removing non-compliant food items from the market places a significant financial burden on exporting countries and food businesses. Therefore, controlling OTA contamination offers potential benefits to both public health and the economy. The implementation of preventive measures and control strategies is crucial for reducing OTA exposure. Various approaches have been introduced to prevent OTA contamination of different food commodities [29]. By combining these strategies, we can effectively reduce the occurrence and frequency of OTA-contaminated food commodities worldwide [30].

In this review, we comprehensively examine the structural properties and metabolites of OTA, its toxicity, analytical methods, relevant legislation, ochratoxigenic fungi, and the occurrence and control strategies pertinent to major food commodities. Furthermore, we shed light on the significant public health and economic ramifications arising from food products contaminated with OTA and discuss prospective approaches for managing emerging OTA hazards. The literature for this review was sourced from the core collection of the Google Scholar online database, covering publications from 2004 to 2024.

## 2 Structural characteristics and properties of OTA

**Fig. 1:**
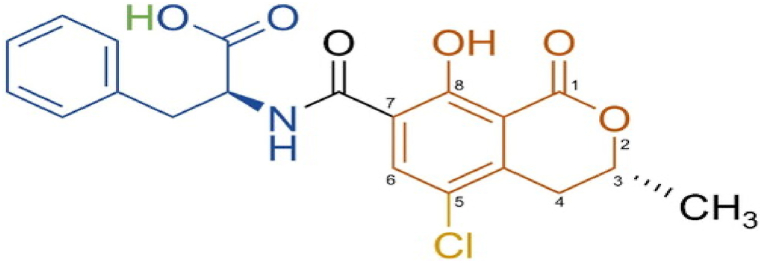
Chemical structure of OTA.

Ochratoxin A (OTA) is characterized by a dihydro-isocoumarin moiety linked to phenylalanine via an amide bond (Fig. 1) [31,32]. Its IUPAC name is *L*-phenylalanine-N-[(5-chloro-3,4-dihydro-8-hydroxy-3-methyl-1-oxo-1*H*-2-benzopyran-7-yl) carbonyl]-(*R*)-isocoumarin [33]. OTA is a white, odourless crystalline solid that displays intense blue fluorescence under alkaline conditions and green fluorescence under ultraviolet (UV) light and acidic conditions [34,35]. It is soluble in polar organic solvent at neutral pH and acid conditions, slightly soluble in water, and insoluble in saturated hydrocarbons [36]. OTA exhibits remarkable stability, with 3 h of high-pressure steam sterilization at 121 °C, although partial degradation occurs even at 250 °C [37]. Complete degradation can be achieved by treating the OTA solutions with excess sodium hypochlorite [33].

## 3 Metabolites of OTA

**Fig. 2:**
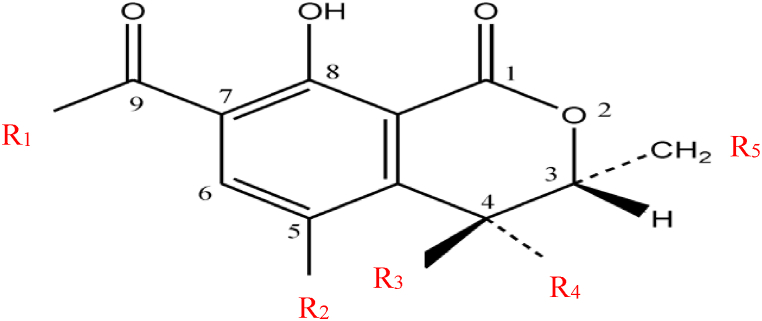
Common structure of OTA-derived metabolites.

Previous studies have indicated that OTA remains largely unchanged, and its toxic effects are believed to be mediated through its metabolism [38]. However, until recently, species-specific differences in OTA metabolism remained unclear. Several metabolites have been characterized *in vitro* and/or *in vivo* across different species. Nonetheless, there are still unknown metabolites that require further characterization. The principal metabolic pathway of OTA involves hydrolysis, hydroxylation, lactone opening, and conjugation. OTA undergoes metabolic transformations leading to several recognized analogs. Notably: (1) ochratoxin B (OTB), the non-chlorinated analog of OTA, which shares a similar structure but lacks the chlorine atom; (2) ochratoxin C (OTC), the ethyl ester of OTA resulting from esterification; (3) ochratoxin α (OTα), an isocoumarin derivative of OTA; (4) ochratoxin β (OTβ), the dechlorinated analog of OTA; and (5) phenylalanine methyl and ethyl ester derivatives [8,39]. The common structural features of these distinct metabolites are depicted in Fig. 2, and Table 1 shows the specific chemical constituents of each metabolite. Additionally, following activation by cytochrome P450 and peroxidase enzyme, OTA generates quinone (OTQ) and hydroquinone (OTHQ) electrophiles and radical species, respectively [25].

**Table 1:** Chemical composition of OTA and its derived metabolites.

Compound	Characteristic chemical constituent				
	R1	R2	R3	R4	R5
OTA	Phenylalanine	Cl	H	H	H
OTB	Phenylalanine	H	H	H	H
OTC	Ethyl ester, phenylalanine	Cl	H	H	H
OTα	OH	Cl	H	H	H
OTβ	OH	H	H	H	H
OTA methyl ester	Methyl ester, phenylalanine	Cl	H	H	H
OTB methyl ester	Methyl ester, phenylalanine	H	H	H	H
OTB ethyl ester	Ethyl ester, phenylalanine	H	H	H	H
4R-Hydroxy-OTA	Phenylalanine	Cl	H	OH	H
4S-Hydroxy-OTA	Phenylalanine	Cl	OH	H	H
10-Hydroxy-OTA	Phenylalanine	Cl	H	H	OH
OTHQ	Phenylalanine	OH	H	H	H
OTQ	Phenylalanine	O	H	H	H

## 4 Toxicokinetics of OTA

Alterations in the concentration of OTA over time coupled with its interactions with biological targets significantly influence its toxicity. Over the years, extensive research has elucidated the toxicokinetics of OTA and its toxicological effects have been well documented. Moreover, understanding the kinetics of OTA, specifically its absorption, distribution, metabolism, and excretion processes, is crucial for understanding it's *in vivo* toxicological impact.

### 4.1 Absorption, distribution, metabolism, and excretion

OTA is primarily absorbed in the small intestine. Animal studies have indicated that both non-ion (OTA^0^) and monoanion (OTA^−^) forms of the toxin are passively absorbed from the stomach and proximal jejunum [40,41]. The extent of absorption varies across species: fish (1.6 %); chicken (40 %); rats and rabbits (56 %); pigs (66 %); and mammals and birds (44–97 %) [37,40,42]. After absorption, OTA is transported to various tissues and organs via the portal system. The brain, fat tissue, and skeletal muscle contain lower OTA levels, whereas the kidney and the liver are recognized as the main target organs [37,40,43].

**Fig. 3:**
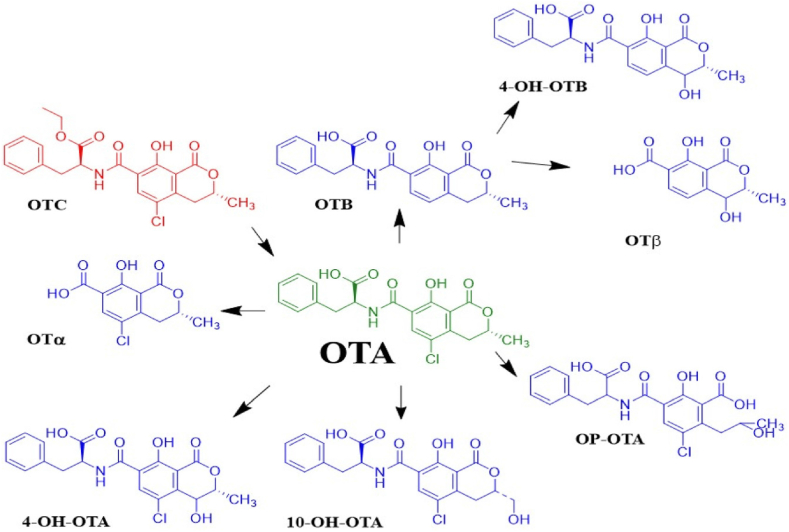
OTA biotransformation occurs via phase I (OTα, OP-OA, 4-OH-OTA, and 10-OH-OTA), phase II (hex/pen-OTA, conjugates of; sulfate, glucuronic acid, and glutathione), dechlorination (OTB), and ethyl esterification (OTC) reactions [37].

OTA undergoes phase I and phase II biotransformation (Fig. 3). Phase I reactions involving cytochrome P450 (CYP450) enzymes includes hydrolysis to ochratoxin α (OTα), lactone-opened ochratoxin (OP-OA), microsomal oxidation to 4-hydroxy ochratoxin A (4-OH-OTA) and 10-hydroxy ochratoxin A (10-OH-OTA) [37]. Additionally, OTA can be transformed into ochratoxin B (OTB) via a dechlorination [44]. Phase II conjugates OTA with glutathione, glucuronic acid, sulfate, and hexose/pentose. OTA elimination occurs through fecal and renal excretion as well as in breast milk [45]. Infants in Chile ingest an average of 12.7 ± 9.1 ng/kg body weight of OTA from breast milk during the first six days after birth [46].

## 5 Toxicity of OTA

OTA, which is frequently encountered in food, poses a significant risk to human health. Although the association between OTA exposure and health risks remains controversial, numerous studies have highlighted its impact on human health. It primarily targets the kidney and has been classified as a possible human carcinogen (Group 2B) [47]. Beyond its carcinogenic potential, OTA exhibits nephrotoxic, hepatotoxic, teratogenic, neurotoxic, genotoxic, and immunotoxic properties [[16], [17], [18], [19], [20], [21]]. OTA's limited solubility in water and its strong affinity for plasma proteins contribute to its unique pharmacokinetic profile. Unlike most xenobiotics, OTA is not readily excreted but rather reabsorbed in the kidney and recirculated, resulting in a prolonged half-life of approximately 5 weeks (35 days) in humans [40]. Several mechanisms underlying the toxicity of OTA have been proposed: (1) lipid peroxidation [48]; (2) protein synthesis inhibition [20,49]; (3) DNA damage [50]; (4) oxidative stress [51,52]; and (5) mitochondrial dysfunction [53,54]. OTA-induced oxidative stress results from the production of reactive oxygen species (ROS). Proximal tubule cells in the kidney are particularly vulnerable, leading to cellular lesions [55]. OTA not only elevates ROS levels but also affects detoxification enzymes via the PXR and AhR pathways [56,57]. Reduced expression of antioxidant-related genes, including the transcription factor Nrf2, compromises cellular defense mechanisms [57]. Paradoxically, OTA may activate antioxidant enzymes by increasing ROS levels and Nrf2 expression [52,58]. Ultimately, OTA-induced oxidative stress contributes to kidney and liver toxicity with redox cycling playing a direct role. The intricate toxicity of OTA involves oxidative stress, impaired cellular defenses, and organ-specific effects. Understanding these mechanisms is crucial for developing effective risk assessment and management strategies.

## 6 Methods of analysis of OTA in agricultural food commodities

### 6.1 Matrix preparation

OTA analysis involves a series of steps, including extraction, clean-up, detection, quantification, and confirmation of identity [25]. While conventional liquid extraction methods are commonly used for OTA determination, solid phase extraction (SPE) has gained prominence, especially for the OTA analysis of agricultural food commodities. Commercially available immunoaffinity columns (IAC) have become popular tools [59]. These columns consist of anti-OTA antibodies and a three-dimensional network specific to the target molecule. IAC significantly enhances OTA analysis by offering several advantages, including clean extracts, improved precision and accuracy, and rapid processing [60]. The primary benefits of IAC are the specific binding of OTA to the antibody and the near-complete removal of matrix interference [61].

### 6.2 Detection techniques

Chromatography plays a pivotal role in mycotoxin analysis, particularly in food safety. Among the various chromatographic techniques, thin layer chromatography (TLC) is an early method that is currently employed for the rapid screening of specific mycotoxins through visual assessment or instrument densitometry [62]. However, recent trends underscore the need for robust and cost-effective technologies that offer high sensitivity and selectivity in a single analytical run [63]. Several chromatographic methods have been developed to address these requirements. For instance, high-performance liquid chromatography (HPLC) coupled with ultraviolet (UV), diode array (DAD), fluorescence (FLD), or mass spectrometry (MS) detectors have gained prominence. Additionally, ultra-high-performance liquid chromatography (UHPLC) and ultra-performance liquid chromatography (UPLC) provide efficient mycotoxin detection [64]. Gas chromatography (GC) coupled with electron capture (ECD), flame ionization (FID), or MS detectors is useful for identifying and quantifying volatile mycotoxins, such as patulin. However, due to the low volatility and high polarity of most mycotoxins, GC analysis often requires derivatization, limiting its application to mycotoxin analysis [65]. The coupling of liquid chromatography with mass spectrometry has significantly advanced OTA analysis. HPLC, combined with mass spectrometric or fluorescence detectors, remains the routine choice for OTA analysis in food, whereas other chromatographic techniques are occasionally used owing to inadequate sensitivity. Among the non-mass spectrometry (MS) methods, high-performance liquid chromatography with fluorescence detection (HPLC-FLD) stands out for quantitative mycotoxin analysis, particularly focusing on OTA [64,65]. HPLC-FLD methods have gained recognition from authoritative bodies, such as the Association of Official Analytical Chemists (AOAC) for assessing OTA levels in cereals. While achieving a sensitivity comparable to liquid chromatography-tandem mass spectrometry (LC-MS/MS), HPLC-FLD is best suited for individual mycotoxins or chemically related groups [66].

Recent advancements include the detection of OTA using HPLC-FLD: (1) detection of maize cereal products, ginseng, and ginger [67,68]; and (2) identification of cereal grains, rye, rice, and corn [63,69,70]. Despite its sensitivity and recovery advantages, HPLC-FLD requires extensive clean-up for accurate OTA detection. However, mass spectrometry (MS) offers distinct benefits over all liquid chromatography (LC) methods for OTA analysis in food. MS provides higher sensitivity, selectivity, and structural information by ionizing molecules and sorting them based on their mass-to-charge ratio (*m*/*z*) [[71], [72], [73]]. Although initially used for single mycotoxin analysis, mass spectrometry (MS) has evolved to simultaneously quantify over 90 mycotoxins in a single run, becoming the preferred method for detecting multiple mycotoxins across a wide range of food samples.

Over the years, rapid diagnostic test kits have played a crucial role in improving food safety. Recently, there has been growing interest in developing on-site test strips specifically designed for detecting major food contaminants, including OTA [74]. These test strips offer rapid and efficient screening, making them valuable tools for ensuring food safety. Lateral-flow devices (LFDs) are commercially accessible tools designed for rapid on-site OTA testing [75,76]. LFDs offer a straightforward single-step process. They incorporate both sample lines and a negative control line on the same strip. The results are semi-quantitative and can be obtained in less than 10 min without the use of specialized equipment. An LFD comprises three essential components: labelled antibodies, porous membranes, and absorbent pads [77]. LFDs operate based on competitive immunoassays. In this mechanism, the labelled antibody acts as a signal reagent. The interaction between the target analyte (such as OTA) and the labelled antibody determines the visual or quantitative results obtained from the LFD [78]. Recently, these devices have been coupled with spectrometric readers to provide quantitative results [79]. Commercially available LFDs can detect various mycotoxins, including OTA. Despite their convenience, LFDs face challenges related to sensitivity, reliability across different matrices, and cost-effectiveness [80]. Furthermore, other rapid diagnostic formats have emerged beyond LFDs. Notably, the initial dipstick assays targeted fumonisin-B1 in corn-based foods, achieving a visual limit of detection of 40–60 μg/kg [81]. Multianalyte dipstick immunoassays for various mycotoxins have been developed, but their sensitivity remains limited. A multiplex dipstick immunoassay specifically detects OTA in rye and rye-based products [82]. In addition, flow-through membranes that operate on the same basic principle as LFDs but may yield less accurate results near the detection limit have emerged [83].

Despite these advancements, rapid strip tests for major mycotoxins have not been widely adopted due to challenges related to sensitivity, cost, and accuracy [84]. Further research and refinement are necessary to enhance their practical utility.

## 7 OTA producing fungi

Fungi exhibit remarkable species diversity compared with other microorganisms, making them ecologically and economically significant. Molecular studies suggests that approximately five million fungal species exist, although only approximately 250,000 species have been formally described [85].

The mycotoxin OTA has been widely reported in various food commodities and products, including cocoa, coffee, cereals, spices, meat, dried fruits, grape juice, wine, and beer [36,86]. Fungal contamination is heterogenous, varies across crops, and is influenced by climatic conditions [87,88]. For example, *Penicillium* species are prevalent in relatively low-temperature regions, whereas *Aspergillus* species dominate in mild and warm climates [89]. The production of OTA by these genera (Table 2) depend on factors such as climatic conditions, substrate water activity, and micronutrient availability [90,91]. Notably, *A. ochraceus* produces more OTA at a water activity (a_w_) of 0.98, regardless of temperature [89]. Conversely, OTA production by *P. verrucosum*, *A. carbonarius*, and *A. ochraceus* increase under optimal condition: 24 °C and 0.95–0.99 a_w_; 15–20 °C and 0.95–0.98 a_w_; and 25–30 °C and 0.98 a_w_, respectively [36,[90], [91], [92]]. Taxonomically, *A*. *ochraceus* belongs to section Circumdati, whiles *A*. *carbonarius* and *A*. *niger* fall within section Nigri [93].

**Table 2:** Ochratoxin A producing fungi in the genera *Aspergillus* and *Penicillium*.

Fungi	Source	Reference
*Aspergillus carbonarius*	Coffee, grapes, red pepper	[96]
*Aspergillus niger*	Maize, beer, coffee, grapes	[96]
*Aspergillus ochraceus*	Green coffee, cereals, soya beans, nuts	[97]
*Aspergillus muricatus*	Peanuts	[98]
*Aspergillus steynii*	Coffee, grapes, barley	[97]
*Aspergillus melleus*	Cereals	[98]
*Aspergillus subramanianii*	Nuts	[98]
*Aspergillus pseudoelegans*	Soil	[98]
*Aspergillus welwitschiae*	Pistachio, walnuts, grapes, raisins	[98]
*Aspergillus flocculosus*	Grapes	[98]
*Aspergillus sclerotioniger*	Coffee	[99]
*Aspergillus sclerotiorum*	Apples	[98]
*Penicillium nordicum*	Cereals	[100]
*Penicillium verrucosum*	Cereals	[100]
*Penicillium radicola*	Potato, carrot	[100]

Misidentification of isolates has led to erroneous reports of OTA-producing fungal speies, and some isolates have undergone reclassification. For instance, many *A. ochraceus* strains have been reclassified as *A. steynii* [36] and the original OTA-producing *A. ochraceus* species is known as *A. westerdijkiae* [94]. Major OTA producers, including *P. verrucosum*, *P. nordicum*, *A. carbonarius*, *A. steynii*, *A. ochraceus*, *A. alliaceus*, and *A. westerdijkiae* are primarily associated with crops during pre-harvest, post-harvest, and storage phases [95].

## 8 Occurrence of OTA in agricultural food commodities and products

Food and agricultural products often contain OTA contamination, posing health risk to consumers [[101], [102], [103], [104], [105]]. Consequently, the global occurrence of OTA in agricultural food commodities is acknowledged as a significant burden [106].

### 8.1 OTA in cocoa

Cocoa, a vital cash crop, serves as a raw material for chocolate production [107]. While many countries cultivate cocoa, approximately 71 % of global production originates in West Africa [31,108]. In the context of cocoa safety, OTA has emerged as a significant mycotoxin. Numerous studies have investigated the OTA contamination in cocoa and cocoa-based products from different regions. Banahene et al. [109] analyzed 520 cocoa bean samples collected directly from farmers' gate. Their findings revealed that 21.7 % of the samples tested positive for OTA with mean concentration ranging from 0.01 to 12.36 μg/kg. Meanwhile, Pires et al. [110] examined Brazilian cocoa and reported an average OTA concentration of 1.2 μg/kg in 123 samples. None of the tested samples exceeded the Brazilian regulatory limit of 10 μg/kg. Similarly, Maciel et al. [111] detected OTA in approximately 18 % (23/130) of Brazilian cocoa samples, with a maximum concentration of 274.9 μg/kg. In Turkey, Kabak [112] surveyed chocolate products and found OTA presence in 46.7 %, 22.8 % and 17.4 % of bitter chocolate, milk chocolate and chocolate wafers, respectively, out 130 total samples. Kedjebo et al. [113] explored Cote d’Ivoire's cocoa, revealing that 32 % of the samples exceeded the OTA threshold of 2 μg/kg. Furthermore, Dano et al. [114] investigated OTA levels at various post-harvest stages (pod opening, fermentation, drying, and storage) in Cote d’Ivoire. Their study identified OTA concentrations ranging from 0.025 to 0.569 μg/kg. In Japan, Aoyama et al. [115] assessed retail cocoa products and found OTA contamination in approximately 97 % (37/38) of the samples, with a maximum concentration of 3.45 μg/kg. Dongo et al. [116] reported 91.5 % ready-to-sell cocoa beans Nigeria tested positive for OTA, with concentrations ranging from 1.0 to 277.5 μg/kg. These findings underscore the importance of monitoring OTA levels in cocoa production and highlight the need for effective control measures to ensure food safety. Research policymakers should continue to address this critical issue to safeguard consumers and the cocoa industry.

### 8.2 OTA in coffee

Coffee is a globally consumed food product and has significant economic importance [117]. However, coffee beans are vulnerable to mycotoxigenic fungi throughout various production stages from harvest to post-harvest, transport, and storage [118]. Among the major mycotoxins, OTA naturally contaminates green coffee beans, roasted coffee, and instant coffee [119]. A survey conducted by Aoyama et al. [115] in Japan revealed OTA presence in approximately 24 % (5/21), 37 % (18/49), 95 % (63/66), and 27 % (11/41) of green coffee beans, roasted coffee beans, instant coffee, and coffee beverages, respectively. Concentrations ranged from 0.40 to 0.76 μg/kg, 0.55–2.75 μg/kg, 0.72–4.23 μg/kg, and 0.026–0.039 μg/kg. Similarly, a survey conducted by Yazdanfar et al. [120] in Iran identified OTA in classic and instant coffee samples ranging from 3.6 to 26.6 μg/kg. Furthermore, a survey conducted in Cambodia by Oeung et al. [121] revealed the presence of OTA in 7.5 % of 40 roasted coffee bean samples with concentrations ranging from 0.19 to 1.12 μg/kg.

### 8.3 OTA in cereals and cereals-based food

Cereals and cereal-based products are staple foods in many countries and play a crucial role in human nutrition. However, like other crops and food items, cereals are susceptible to contamination by ochratoxigenic fungi, particularly under specific climatic and environmental conditions [122].

Studies on dietary exposure to OTA has highlighted cereals and cereal-based foods as significant contributors to OTA intake [123]. Notably, the estimated OTA exposure through these food sources is substantial for both Canadian populations: over 75 % for one-year-olds and more than 60 % for males aged 31–50 [124]. In a study involving 64 samples, 41 % tested positive for OTA, with the highest concentration reaching 1.1 μg/kg [12]. Similarly, a comprehensive investigation conducted between 2009 and 2014 in the Canadian retail market revealed the presence of OTA in 3657 out of 6857 grain-based and non-grain-based food products, with concentrations ranging from 0.04 to 631 μg/kg. Notably, wheat, oats, milled products from other grains (such as rye and buckwheat), and corn-based products have emerged as the primary sources of OTA exposure in the Canadian population [125]. Furthermore, a survey conducted in Turkey by Kabak [126] identified OTA in 38 % of 24 breakfast cereals samples, with concentrations ranging from 0.172 to 1.840 μg/kg. Additionally, 17 % of 24 cereal-based baby food samples contained OTA at levels between 0.122 and 0.374 μg/kg. In Morocco, Zinedine et al. [127] reported that 90 % of 20 rice samples positive for OTA with concentrations spanning from 0.02 to 32.4 μg/kg.

### 8.4 OTA in wine and beer

Global surveys across different countries have consistently highlighted OTA as a significant natural contaminant of beer and wine [128]. Given its adverse effects on human health, OTA has garnered considerable attention as a contaminant in alcoholic beverages. Cao et al. [129] reported OTA incidence of OTA in 4 out of 30 samples of beer, red wine, and grape juice. Similarly, a Tunisian study by Lasram et al. [130] found OTA contamination 85 % of wine samples and 45 % of beer samples, with concentrations ranging from 0.09 to 1.5 μg/L and 0.04–0.35 μg/L, respectively. Bertuzzi et al. [131] reported that 67.9 % of 106 beer samples were OTA-positive, with concentrations ranging between 0.002 and 0.189 μg/L. Other investigations also detected OTA in beer, with levels ranging from 0.03 to 0.25 μg/L [132,133]. Table 3 summarizes the occurrence of OTA in various food commodities and products worldwide.

**Table 3:** OTA occurrence in food commodities and products from various countries.

Country	Food commodity/Product	% contaminated	OTA (μg/kg)	Reference
Algeria	Wheat	76.54 % of 81	0.84–34.75	[134]
	Cereal-based foods	16.9 % of 71	0.06–0.2	[135]
Brazil	Cocoa beans	22.8 % of 123	0.25–7.2	[110]
	Fermented coffee	21.4 % of 14	<0.64–0.87	[15]
Canada	Wheat	2.2 % of 250	6.8–22.6	[136]
	Cocoa and chocolate	100 % of 60	0.05–7.8	[137]
China	Pasteurized milk	25.8 % of 120	>0.049–18.8	[138]
	Maize	1.6 % of 426	0–5	[139]
Croatia	Dry cured-meat	19.2 % of 250	0.24–4.81	[140]
Czech	Beer	81 % of 132	0.001–0.195	[141]
Egypt	Barley	20 % of 15	1.13–2.15	[142]
Iran	Dried grapes	57.5 % of 23	0.16–8.4	[143]
Italy	Cheese	26.3 % of 57	1.7–7.2	[144]
	Milk	36.4 % of 33	<0.3–3.0	[145]
	Salami	12.8 % of 172	0.07–5.66	[146]
	Wine	71.9 % of 57	0.13	[147]
Morocco	Dried fruits	17.1 % of 210	0.8–99.1	[148]
Pakistan	Maize	71 % of 46	2.14–214.0	[11]
Portugal	Beer	10.6 % of 84	<0.43–11.25	[149]
	Coffee	25 % of 6	1.45–10.31	[9]
	Rice	2 % of 36	1.9–2.2	[150]
Serbia	Breakfast cereals	33.7 % of 136	0.07–3.0	[13]
	Dry wine	52.2 % of 113	0.026	[14]
Spain	Beer	20 % of 40	0.24–54.76	[151]
Turkey	Dried figs	8 %	–	[152]
Tunisia	Sorghum	37.5 % of 64	1.04–27.8	[153]
Uganda	Cereals	8.3–100 % of 105	0.1–16.4	[10]
USA	Raisins	93 % of 40	0.06–11.4	[154]
	Barley	6 % of 60	0.16–185.24	[155]
	Wheat	13 % of 58	0.17–14.94	[155]

## 9 Public health and economic implications of OTA

OTA has been associated with several adverse health effects. It has been linked to cancer, immune system toxicity, kidney disease, and birth defects [156,157]. Notably, in Balkan nations, OTA has been identified as a significant factor contributing to human endemic nephropathy owing to its nephrotoxic properties [158,159]. Humans are commonly exposed to OTA through the consumption of contaminated foods. However, chronic effects may manifest when low doses are ingested over an extended period [160].

Various staple foods including cereals, coffee, cocoa and cocoa-derived products, grape juice, wine, and beer are consumed globally. Unfortunately, certain fungal species from the genera *Aspergillus* and *Penicillium* can produce OTA in these foods, leading to toxic effects on humans upon consumption. Contamination of the food product occurs at different stages, from production to storage of the final product [161].

Animal-derived products, such as milk, also contribute to OTA exposure in humans [162]. Monitoring food ingredients used in animal feed production is crucial to prevent cattle exposure to OTA, which can be excreted in milk and subsequently consumed by humans [163]. The presence of OTA in milk poses significant risk, particularly for infants and children, as it can suppress the immune system and potentially lead to cancer. Consequently, OTA is a serious health concern.

Beyond its health implication, OTA contamination has substantial economic and commercial repercussions [164]. Reports indicates that mycotoxins, including OTA, are detectable in approximately 60–80 % of food samples [1].

OTA-contaminated products can result in substantial costs for economies that are heavily reliant of food exports. For instance, cocoa serves as a critical economic backbone in West African sub-regions such as Ghana [108]. Similarly, coffee significantly contributes to export-generated income in certain countries. Thus, contamination of these vital food crops could have severe consequences for their economies [165]. Vigilant control measures are essential to mitigate the impact of OTA on both health and economic fronts.

## 10 Regulation of OTA and its impact on food safety and trade

The regular consumption of OTA poses significant health risks to both human and animals. To mitigate these risks, regulatory frameworks have established maximum allowable thresholds for OTA in various food commodities and products [166]. While these regulations enhance safety, they can also hinder trade from regions susceptible to contamination, potentially diminishing the economic value of the affected goods. Socio-economic factors significantly influence mycotoxin contamination. Inadequate government policies contribute to the presence of mycotoxins in food [30]. Approximately 90 countries worldwide regulate OTA levels to safeguard public health. However, these regulations also affect nations exporting contaminated products. Striking the balance between financial and regulatory benefits is crucial. Even in countries with established OTA regulations, uninspected food commodities remain a risk, impacting global trade and public health [167]. The permissible OTA limit for food intended for human consumption varies globally, ranging from 0.5 to 20 μg/kg. The EU has implemented stringent regulatory limits for OTA in various food items. For example, cereals, roasted coffee, and wine have maximum levels of 3, 5, and 2 μg/kg, respectively. Brazil's regulatory agency ANVISA has established tolerable levels of mycotoxins, including OTA. However, new regulations are progressively tightening the standards. Interestingly, the FDA lacks specific directives regarding the occurrence of OTA in foods in the US. Instead, the US relies on norms set by other regions for OTA regulation. Furthermore, the Codex Alimentarius Commission, through the Joint FAO/WHO Food Standards Programme Codex Committee on Contaminants in Food and Health Canada, has proposed regulatory limits on a range of food products.

In summary, the EU maintains stricter OTA limits, whereas Brazil's regulations are generally more permissive. Understanding these variations is essential to global trade and public health. Table 4 presents a concise comparison of the acceptable OTA limits across food products in the EU, Brazil, and Canada.

**Table 4:** Regulatory limits for the presence of OTA in various food commodities and products.

Region/Organization	Food commodity/Product	OTA limit (μg/kg)
EU	Cocoa powder	3.0
	Coffee	3.0–5.0
	Grape, grape juice and wine	2.0
	Dried fruits	2.0–8.0
	Cereals and cereals-derived products	3.0–5.0
	Cereal-derived food for infants	0.5
Brazil	Cocoa beans	10.0
	Coffee	10.0
	Cocoa and chocolate	5.0
	Dried fruits	10.0
	Grape, grape juice, and wine	2.0
	Maize and maize-derived foods	20.0
	Cereals and cereals-derived foods	10.0
	Cereal-derived food for infants	2.0
Canada	Raw/unprocessed cereal grains	5.0
	Wheat bran	7.0
	Cereals-based foods for infants	0.5
	Grains for direct consumption	3.0
	Derived cereal products	3.0
Codex	Raw wheat	5.0
	Barley	5.0
	Rye	5.0

## 11 Prevention and control of OTA in agricultural food commodities and products

Achieving OTA-free food and food products presents several challenges. However, mitigating mould and subsequent mycotoxin contamination in the various agricultural food items is both feasible and serve as an effective control measure [30]. These challenges occur at different stages: pre-harvest (including crop production and growth) [168] and post-harvest (encompassing harvest, transport, storage, and processing) [169,170]. Notably, addressing mould infestation and detoxification of the toxin before harvest has significant potential [171,172].

### 11.1 Physical control

Physical control strategies encompass a range of measures aimed at mitigating mycotoxin contamination of crops and products. These strategies include mould prevention, decontamination, detoxification, cleaning, sorting, and toxin monitoring. A critical aspect of mycotoxin management involves preventing the invasion of ochratoxigenic fungi into fields, as this significantly affects the subsequent production of OTA in food crops [173]. Effective practices for OTA control include the timely cultivation and harvesting of crops under suitable conditions, removal of inoculum sources (such as decaying agricultural residues), and crop rotation [174]. Additionally, minimizing direct contact between crops and soil is recommended to prevent the spread of mycotoxins [175]. During food processing, heat treatment at elevated temperatures can reduce mycotoxin levels [176]. For instance, exposure to temperatures exceeding 150 °C leads to an 84 % reduction in mycotoxins [177]. However, high-temperature treatments may compromise the nutritional quality. Conversely, mild temperature conditions (within the typical food treatment range of 80–121 °C) may be less effective due to mycotoxin heat resistance [178].

Another approach involves ionizing radiation, specifically, gamma rays. The Manual of Good Practices for Food Irradiation, jointly developed by the International Atomic Energy Agency (IAEA) and the Food and Agriculture Organization of the United Nations (FAO), supports this method [179]. Vita et al. [179] demonstrated a 23.9 % reduction in OTA contamination in almonds with a gamma ray dose of 15 kGy. Electron-beam irradiation at 50 kGy also achieved an OTA reduction exceeding 67 % in naturally contaminated corn [180]. However, safety concerns related to mutagenesis and potential damage to nutritional content warrant further investigation and public awareness [75].

### 11.2 Chemical control

Various chemical agents have shown efficacy in decontaminating and mitigating the physiological effects of mycotoxins [181]. Acids such as citric, lactic, tartaric, acetic, hydrochloric, and succinic acids have been employed in the food production industry [182]. Alkaline substances such as ammonia and sodium bicarbonate, as well as salts (sodium bisulfite and sodium chloride) and oxidizing agents (sodium hypochlorite and hydrogen peroxide), have also been utilized, achieving an approximate 90 % decontamination rate [183]. Specifically, sodium bicarbonate (NaHCO₃) and potassium carbonate (K₂CO₃) have been identified as effective decontaminants against ochratoxin A (OTA) in cocoa shells under varying conditions of time, pressure, and temperature [184]. However, it is important to note that these agents do not inhibit fungal growth at very low doses, and may stimulate OTA production. Additionally, the use of chemical fungicides could potentially induce resistance to other introduced substances, thereby reducing post-application pathogen growth [185]. Furthermore, the practical application of existing chemical decontamination methods for OTA-contaminated agricultural food commodities and products is constrained by health and safety considerations. Accumulation of toxic chemicals poses risks to both human health and the environment and can also impact the nutritional quality of food. Consequently, strict adherence to regulations is essential to ensure that pesticide residues in food commodities remain below the maximum limits established by prevailing legislation.

### 11.3 Biological control

Biological control strategies are employed to inhibit fungal growth and ochratoxin A (OTA) formation in food commodities [186,187]. For instance, Podgorska-Kryszczuk et al. [188] demonstrated the effectiveness of *Aureobasidium pullulans* and *Saitozyma podzolicus* as a biocontrol agent against *Aspergillus parasiticus* and *Aspergillus ochraceus* in bread, thereby reducing OTA contamination. Alsalabi et al. [189] also reported the inhibitory effects of *Burkholderia cepacia* bacteria on ochratoxigenic fungi and OTA production in animal feed. Additionally, the inoculation of certain microbes, such as *Saccharomyces* and *Lactobacillus*, into OTA-contaminated food products restricts the spread of ochratoxigenic strains [190]. Notably, research has shown that OTA reduction occurs due to factors such as growth restriction of ochratoxigenic fungi, depletion of essential nutrients required for OTA synthesis by antagonistic fungi, and enzymatic breakdown of OTA by atoxigenic fungi [191]. Natural agents such as essential oils have been utilized as preservatives in the food industry to combat pathogenic and spoilage microbes [192]. Essential oils exhibit strong antimicrobial properties and have been investigated for their ability to prevent fungal growth and detoxify OTA. Khoury et al. [193] observed reductions in OTA levels (ranging from 25 % to 80 %) in a synthetic grape medium when using sage and melissa essential oils. Similarly, Koteswara et al. [194] reported that mycotoxin degradation by essential oils, including neem oil and eucalyptus, led to a reduction in OTA. Aldred et al. [195] found that essential oils such as cinnamon oil, thyme, and clove inhibited the growth of certain Aspergillus spp. In a study involving spiked cocoa powder and beverages, essential oils and an aqueous extract of Aframomum danielli effectively reduced OTA levels. Notably, Daniellin™ eliminated OTA from a non-alcoholic cereal-based drink (kunu-zaki), as reported by Adegoke et al. [196]**.**

## 12 Future directions in management of OTA in agricultural food commodities and products

The presence of Ochratoxin A (OTA) in agricultural food commodities and products poses a significant threat to food safety, public health, and the economy. Pragmatic and multifaceted control and prevention measures are essential to address this issue. For instance, ongoing advancements in sensitive and rapid detection methods, including advanced chromatography techniques, immunoassays, and molecular assays, have enhanced OTA surveillance and monitoring globally. At the agricultural level, improved agronomic practices for pest control, such as the utilization of biocontrol agents from local sources, can help reduce mould and OTA contamination. Adapting agricultural practices and storage conditions to changing climate patterns is crucial to prevent OTA contamination. Implementing effective regulations and guidelines based on OTA occurrence and exposure data, along with collaboration among stakeholders (including government agencies, food producers, and research institutions) further contributes to successful OTA management.

## 13 Conclusion

Ochratoxin A (OTA) poses a significant threat to both human and animal health. Its impact extends beyond health and affects agriculture, related industries, and the economy. Consistent reports have underscored the widespread contamination of essential foodstuffs and widely traded products with OTA. Unfortunately, the complete elimination of OTA formation and contamination in food remains challenging due to the ubiquity of OTA-producing fungi and their resilience to standard food processing temperatures. Consequently, the OTA levels in agricultural products frequently surpass regulatory limits. To address this critical issue, robust prevention, control, and detoxification strategies for OTA in food products are imperative. Implementing these measures will not only reduce contamination rates, but also contribute to the reduction of ochratoxicosis, ensuring food safety, security, and global trade. Efficient monitoring of OTA and public education can further mitigate contamination risks.

**Table:** Abbreviations

OTA	Ochratoxin A
IARC	International Agency for Research on Cancer
NTP	National Toxicology Program
JECFA	Joint FAO/WHO Expert Committee on Food Additives
PTWI	Provisional Tolerable Weekly Intake
EFSA	European Food Safety Authority
EU	European Union
IUPAC	International Union of Pure and Applied Chemistry
PXR	Pregnane X Receptor
AhR	Ary Hydrocarbon Receptor
ROS	Reactive Oxygen Species
Nrf2	Nuclear factor-erythroid factor 2-related factor 2
DNA	Deoxyribonucleic acid
IAC	Immunoaffinity column
USA	United States of America
ANVISA	Agencia Nacional de Vigilancia Sanitaria
FDA	Food and Drugs Authority
IAEA	International Atomic Energy Agency

## CRediT authorship contribution statement

**Joel Cox Menka Banahene:** Writing – review & editing, Writing – original draft, Conceptualization. **Isaac Williams Ofosu:** Writing – review & editing. **Bernard Tawiah Odai:** Writing – review & editing. **Herman Erick Lutterodt:** Writing – review & editing. **Paul Ayiku Agyemang:** Writing – review & editing. **Williams Otoo Ellis:** Writing – review & editing.

## Ethical statement

Not applicable.

## Data availability

Data included in this article have been referenced.

## Declaration of competing interest

The authors declare that they have no known competing financial interests or personal relationships that could have appeared to influence the work reported in this paper.
